# Prognostic value of Goseki histological classification in adenocarcinoma of the cardia

**DOI:** 10.1038/sj.bjc.6600663

**Published:** 2003-02-10

**Authors:** M G Fontana, M La Pinta, D Moneghini, V Villanacci, F Donato, G Rindi, S Paparini, C Baronchelli, G Bertoli, P Alquati

**Affiliations:** 1 Department of Surgery, University of Brescia and Spedali Civili, P. le Spedali Civili 1, 25123, Brescia, Italy; 2 Department of Pathology Service, University of Brescia and Spedali Civili, P. le Spedali Civili 1, 25123, Brescia, Italy; 3 Chair of Hygiene, University of Brescia and Spedali Civili, P le Spedali Civili 1, 25123, Brescia, Italy; 4 Department of Surgery Cremona Hospital, V le Concordia 1, 26100 Cremona, Italy; 5 Pathology Service Cremona Hospital, V. Ie Concordia 1, 26100 Cremona, Italy

**Keywords:** Goseki classification, adenocarcinoma of the cardia, prognostic value

## Abstract

Various histologic classification systems have been proposed as prognostic factors for gastric cancer. We assessed the prognostic value of Goseki classification as well as the TNM staging system, histological tumour grading, Lauren, WHO, Goseki and Siewert classifications in 100 patients with cardia carcinoma undergoing curative surgery. Two patients were lost at follow-up. The median time of follow-up in the remaining patients was 32.9 months after surgery (range: 0.1–142.1 months). No differences in survival rates were observed according to tumour grading, Lauren or WHO histologic or Siewert topographical classification. No differences were found according to Goseki classes, when considering either the mucin content of the carcinoma (types I and III *vs* II and IV) or the differentiation grade (types I and II *vs* III and IV). Multivariate analysis showed that the only lymph node positivity was a significant predictor of survival: 7.2% of patients with, but 41.5% of those without nodal involvement were alive after five years (*P*=0.0001). In conclusion, we found no prognostic role for Goseki or the traditional histological indexes, while the TNM staging system and particularly lymph node positivity were the main predictors of survival in patients with cardia adenocarcinoma.

Gastric cancer is the second most common neoplasm in the world after lung cancer, with about 800 000 new estimated cases per year (Parkin *et al*, 1999). While an overall decline in gastric cancer mortality has been observed in the USA and the European Union since the 50's (Nomura, 1996; Levi *et al*, 1999), a rising incidence of cardia adenocarcinoma has been observed in the same areas (Devesa *et al*, 1998; Botterweck *et al*, 2000), although some findings suggest that these figures may be inflated by some degree of misclassification and increased diagnostic awareness (Ekstrom *et al*, 1999). However, adenocarcinoma of the cardia has different characteristics compared to adenocarcinoma arising in the other sites of the stomach: the male–female ratio is higher, the ethnic background is different, a family history is less common, a past history of chronic heartburn or duodenal ulcer is more frequent, and there is some evidence of an association with tobacco smoking and alcohol drinking (MacDonald and MacDonald, 1987). Furthermore, cardia carcinoma shows a greater tendency to proceed to deep wall penetration, is more often associated with lymph node involvement and has a poorer prognosis than the cancer of other gastric regions (Husemann, 1989; Maehara *et al*, 1991; Ohno *et al*, 1995; Kajiyama *et al*, 1997; Pinheiro *et al*, 1999). Taken together, these findings support the hypothesis that cardia adenocarcinoma may be a biologically distinct disease when compared to adenocarcinoma arising in other stomach sites.

The histological classification systems commonly used for gastric cancer (Lauren, 1965; Ming, 1977; Oota and Sobin, 1977) have also been applied to adenocarcinoma of the cardia. However, follow-up studies on the prognostic value of these indexes in patients with cardia adenocarcinoma have provided conflicting results (Ribeiro *et al*, 1981; Husemann, 1989; Maehara *et al*, 1991; Jakl *et al*, 1995; Ohno *et al*, 1995; Kajiyama *et al*, 1997; Caldas *et al*, 1999; Pinheiro *et al*, 1999). As a consequence, new classification systems have been suggested as prognostic factors. A grading system that combines intracellular mucin content and tubular differentiation has been recently proposed by Goseki *et al* (1992). According to the Goseki classification, four grades of histologic features were defined: group I) – well-differentiated tubules, poor intracellular mucus; group II) – well-differentiated tubules, rich intracellular mucus; group III) – poorly differentiated tubules, poor intracellular mucus; group IV) – poorly differentiated tubules, rich intracellular mucus. The high reliability (inter- and intra-observer agreement) of this classification system has been demonstrated by other research groups (Dixon *et al*, 1994; Songun *et al*, 1999). Two recent studies found that the Goseki histological classification was predictive of survival in patients with gastric cancer (Martin *et al*, 1994; Songun *et al*, 1999), although two other studies did not (Guglielmi *et al*, 1997; Roy *et al*, 1998), and thus the prognostic value of the system remains controversial. To our knowledge, the Goseki histologic classification has so far not been applied in patients with cardia adenocarcinoma.

The aim of this study was to investigate the prognostic value of Goseki classification compared with other histologic indexes used for gastric cancer such as the conventional tumour grading (Oota and Sobin, 1977), Lauren classification (Lauren, 1965), WHO classification (Oota and Sobin, 1977), TNM staging and Siewert topographical classification (Siewert and Stein, 1998) in a consecutive series of patients with cardia carcinoma undergoing potentially curative resection.

## PATIENTS AND METHODS

Consecutive patients with carcinoma of the cardia who underwent potentially curative surgical resection in the Department of Surgery of the University of Brescia and in the Department of Surgery of the main hospital in Cremona between 1st January 1990 and 31st December 1999 were enrolled prospectively. Among them, 58 underwent total, 33 total enlarged and 9 superior polar gastrectomy.

The patients' age and sex were included in the analysis. Three categories of age were considered: <60 years, 60–90 years and >70 years. Neoplasms were staged using the pathologic TNM system (Hermanek and Sobin, 1992). Tumour localization was identified according to Siewert classification (Siewert and Stein, 1998): *type I*, adenocarcinoma of the distal oesophagus; *Type II*, carcinoma of the cardia arising from the epithelium of the oesophagogastric junction; *Type III*, subcardial gastric carcinoma infiltrating the oesophagogastric junction. The histological specimens were classified according to the following systems: tumour grading, Lauren (1965), WHO (Oota and Sobin, 1977) and Goseki *et al* (1992) classifications.

The end of the follow-up period was 31st October 2001. Survival rates were calculated from the date of enrollment in the study (day of surgical operation) up until the end of follow-up or death. Survival curves and confidence intervals were calculated according to the method of Kaplan and Meier (1958). Comparisons among groups were performed using common statistical tests for proportion analysis and the log-rank test for univariate analysis. Cox's (1972) regression models were fitted to evaluate the prognostic significance of the TNM staging system, tumour cell differentiation, Lauren, WHO and Goseki histological classification of the neoplasm, Siewert classification, and patients age and sex, adjusting the effect of any factor for each other. The prognostic value of the Goseki histologic system was further investigated among patients with less advanced neoplasms according to TNM classification. Statistical tests were two-sided and a *P*-value of 0.05 was used for rejecting the null hypothesis. All the analyses were performed using the STATA statistical program for a personal computer (Stata Statistical Software: Release 7.0. College Station, Stata Corporation, TX, USA).

## RESULTS

A total of 100 patients, 76 males and 24 females, of mean age 66.3 years (standard deviation (s.d.): 9.3 years; range: 41–86 years) underwent curative resection for adenocarcinoma of the cardia and were enrolled in the study. The main histopathological findings of the tumours and the corresponding 5-year survival rates are reported in Table 1

**Table 1:** Distribution of patients with carcinoma of the cardia (*n*=100) according to Siewert topographical and various histological classification systems and 5-year survival rate (%)

**Classification of tumours**	**No.**	**5-year survival rate (%)**
Lauren classification		
Intestinal	83	24.1
Diffuse	13	6.3
Mixed	3	0
		
WHO classification		
Papillary	49	20.8
Tubular	7	42.9
Mucinous	9	11.1
Signet ring cell	18	23.5
Undifferentiated	17	17.7
		
Grading		
Well differentiated	9	55.6
Medium differentiated	48	19.2
Poorly differentiated	43	16.7
		
Goseki classification		
Type I	34	26.5
Type II	33	18.8
Type III	20	26.3
Type IV	13	7.7
		
TNM staging		
T		
T1	20	52.6
T2	45	20.5
T3	22	4.6
T4	13	7.7
		
N		
NO	43	41.5
N+	57	7.0
		
Stage		
Stage I	39	43.2
Stage II	25	8
Stage III	35	8.6
Stage IV	1	0
		
Siewert classification		
Type I	5	40
Type II	54	19.2
Type III	41	22

. Most neoplasms were of the intestinal types according to Lauren classification, papillary or undifferentiated cancers according to the WHO classification, medium or poorly differentiated according to the grading system, and Goseki's types I or II. According to TNM classification, most neoplasms were at an advanced stage, with only a minority of neoplasms in Tl or N0 or stage I. With respect to topographical characteristics, 54% of tumours were within 1 cm oral and 2 cm aboral of the gastroesophageal junction (type II according to Siewert's classification), 41% were subcardial (types III) and only 5% were in the distal oesophagus (types I).

Two patients were lost at follow-up. Among the 98 who completed the follow-up, 68 died, 7 of whom within 30 days after surgery owing to complications. Among the 30 patients who were still alive at the end of the follow-up, 18 survived at least 5 years after surgery (18.4% of the total who completed the follow-up). As shown in Table 1, the 5-year survival rates showed substantial variations according to TNM staging (from 43.2% in stage I to 8.6% in stage III) and grading (from 55.6 to 16.7%), and less relevant differences according to the other factors. The median survival time was 32.9 months (range: 0.1–142.1 months); it was longer in females (59.2 months) than in males (24.9 months) (*P*=0.03) but did not vary with age (59.2 months in patients aged less than 60 years, 45.1 months in those aged 60–69 and 44.0 months among those older than 70 years; *P*=0.4).

**Figure 1:**
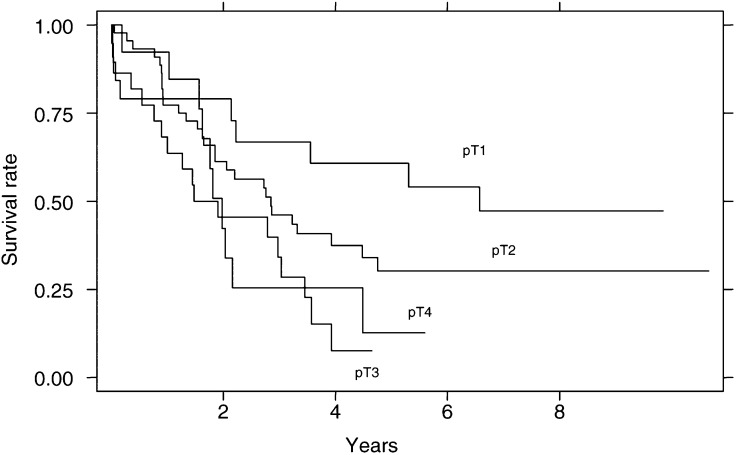
Survival curves according to T stage.

**Figure 2:**
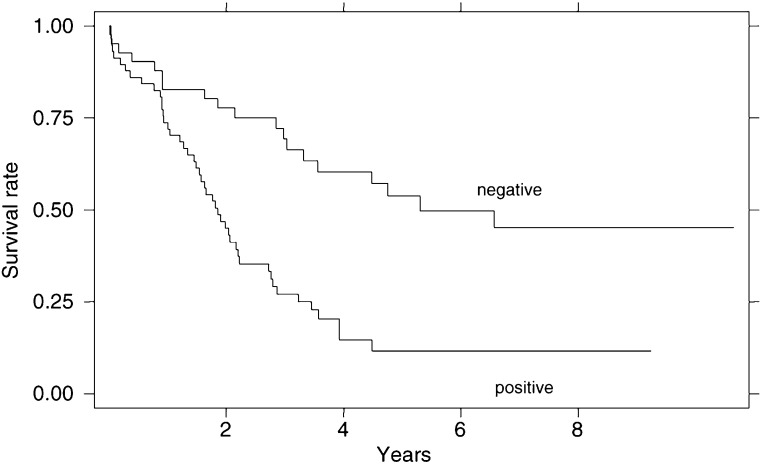
Survival curves according to lymph node involvement.

**Figure 3:**
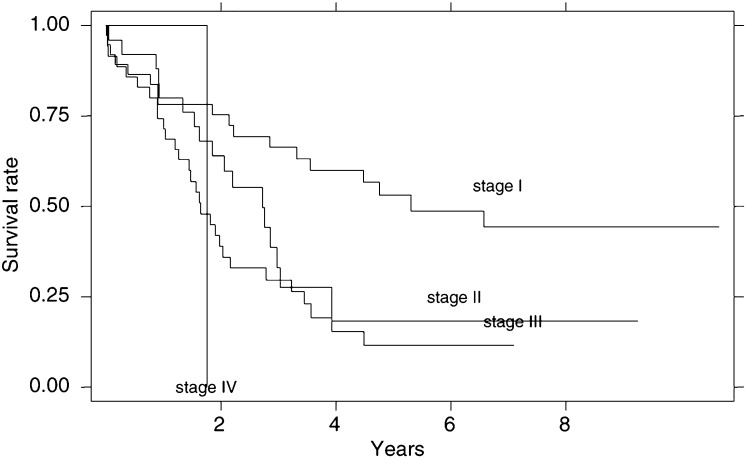
Survival curves according to TNM stage.

The local infiltration depth (T) and nodal involvement (N) were both of prognostic value in univariate analysis. Survival curves according to pathologic T (pT stage) (Figure 1

) showed a better survival of patients with Tl stage tumours than those with more advanced cancer (T2–T4) (median survival times of 71.6 and 25.4 months, respectively, *P*=0.04), with no differences among patients with T2, T3 or T4 stage tumours. After 5 years, 52.6% of patients with Tl cancer but only 11.4% of those with T2–T4 cancer were still alive (*χ*^2^-test: *P*=0.0003). A significant difference in survival was also found according to lymph node involvement (Figure 2

): 41.5% of patients with lymph node negative cancer but only 7% of those with lymph node positive cancer were alive after 5 years (*χ*^2^ -test: *P*=0.0001). The TNM staging system was an independent prognostic variable: stage I showed a median survival of 60.8 months, stage II of 31.7 months and stage III of 19.3 months, with highly significant differences in survival curves (*P*=0.001) after excluding the single patient with a stage IV neoplasm (Figure 3

).

According to conventional tumour grading, survival curves showed differences between patients with Gl grade compared to the others, although they were not significant because of the small number of deaths in the former group (*n*=3) (*P*=0.5) (data not shown). According to the Lauren classification, neoplasms of the intestinal type showed a longer median survival time (33.0 months) than the diffuse (21.8 months) and mixed types (26.5 months), although the differences were not significant (*P*=0.7) (data not shown). Again, no difference was observed in survival according to the WHO classification: the median survival time observed was 24.4 months for papillary adenocarcinoma, 44.4 months for tubular cancer, 32.7 months for mucinous cancer, 28.1 for signet ring neoplasm and 27.0 (*P*=0.9) for poorly differentiated cancer (data not shown). Similarly, no statistically significant difference in survival was found between types I, II and III tumours according to the Siewert topographical classification, the 5-year survival rate being 40, 17.3 and 17.1%, respectively (data not shown).

**Figure 4:**
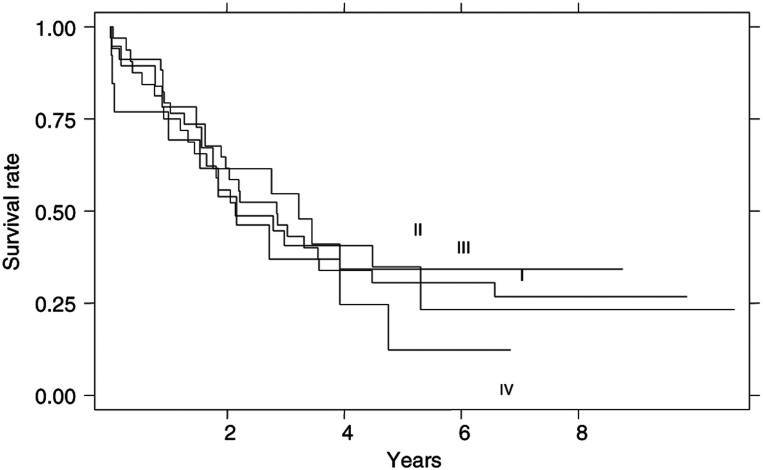
Survival curves according to Goseki classes.

No differences in survival were observed for the different Goseki classes (Figure 4

): median survival times were 32.0 months for types I, 25.0 months for type II, 36.4 months for type III and 23.7 months for type IV (*P*=0.9). In order to assess the prognostic value of the mucin content of the carcinoma, we compared Goseki types I and III with Goseki types II and IV. No significant difference was observed: the median survival time of the ‘poor intracellular mucus’ groups (types I and III) was 33.7 months and that of the ‘rich intracellular mucus’ groups (types III and IV) was 25.3 months (*P*=0.5). To investigate the role of the differentiation grade in Goseki classification, well-differentiated cancer (types I and II) and poorly differentiated cancer (types III and IV) were grouped and compared. Again, no statistically significant difference in survival time was observed (*P*=0.7).

We investigated the role of each variable adjusting for the others (sex, age, TNM staging system, histological tumour grading, and Lauren, WHO, Goseki and Siewert classifications) fitting Cox's proportional hazards models. Multivariate analysis showed that only lymph node positivity was a statistically significant predictor of survival, with a hazard ratio of 3.2 (95% confidence interval: 1.8–5.8) for patients with positive nodes compared to patients with negative nodes. Sex and age were not associated with survival when including TNM staging. Furthermore, the T stage was strongly associated with lymph node positivity, and the independent role of each of them could not be assessed properly. In fact, only two of 57 (3.5%) patients with lymph node involvement, but 18 of the 43 (41.9%) without it, had a more than Tl stage. A stratified analysis according to age categories and sex did not reveal any substantial differences: lymph node positivity remained the only significant predictor of survival in each analysis. Finally, subgroup analysis was performed for Goseki classification according to patients' sex and age and TNM staging and for Siewert classification, but no association with survival was found. A separate analysis showed no difference in survival rates according to Goseki classification when restricted to the 54 patients with Siewert types n cancer only.

## DISCUSSION

The adenocarcinoma of the cardia generally has a low curative resection rate and a poor prognosis, worse than carcinoma of the other regions of the stomach, mostly because of the more advanced stage of the disease at diagnosis (Husemann, 1989; Maehara *et al*, 1991; Ohno *et al*, 1995; Kajiyama *et al*, 1997; Pinheiro *et al*, 1999). The 5-year survival rate in resected cases has been found to be 20% or less (Graham *et al*, 1998).

Among the many characteristics investigated, only the TNM stage consistently proved to be of prognostic value (Maehara *et al*, 1991; Kajiyama *et al*, 1997; Graham *et al*, 1998). Of the newer systems proposed, the Goseki classification is one of the most promising prognostic factors. It is based on two cancer features: tubular differentiation and the degree of mucus production by the tumour cells. Previous reports have suggested that mucus production could be of greater importance than tubular differentiation in the assessment of prognosis (Goseki *et al*, 1992; Martin *et al*, 1994; Songun *et al*, 1999). Mucins are heavily glycosylated glycoproteins that are the main components of the mucous viscous gel covering the surface of epithelial tissues. Changes in the expression levels and glycosylation patterns of mucins have been associated with several diseases, including carcinomas (Sakamoto *et al*, 1997; Reis *et al*, 1999). Alteration in mucin expression has been reported (Sakamoto *et al*, 1997) and such abnormality was proposed as a potentially informative molecular marker of increased risk of malignant transformation in intestinal metaplasia (Reis *et al*, 1999). In 1971, Paile first reported a significantly poorer survival rate in patients with mucus-rich tumours (Paile, 1971), and two later reports demonstrated that signet ring carcinomas, which are rich in intracellular mucus, have a worse prognosis than well-differentiated carcinomas sitting in lakes of extracellular mucus (Brander *et al*, 1974; Ishii *et al*, 1981).

The prognostic value of Goseki classification has been evaluated in only a few studies in patients with gastric cancer with contradictory results (Martin *et al*, 1994; Dixon *et al*, 1994; Guglielmi *et al*, 1997; Roy *et al*, 1998; Songun *et al*, 1999), although some authors have suggested selecting patients for adjuvant therapy after curative resection of cancer according to the Goseki classification (Martin *et al*, 1994).

We aimed to assess the prognostic value of Goseki histological classification compared to other histological and Siewert topographical classification systems in patients undergoing curative surgery for cardia adenocarcinoma. Overall, this study confirmed a poor prognosis for patients with cardia adenocarcinoma, with only 18.4% surviving for 5 years after surgery. We enrolled a relatively small number of patients with a complete follow-up (*n*=98), which means the study did not have much statistical power to show significant differences in survival according to various factors. However, this is the first study assessing the role of Goseki and other prognostic factors in the survival of patients with cardia carcinoma, which is usually considered a clinical entity distinct from gastric carcinoma (Husemann, 1989), and it should therefore be regarded as tentative.

We did not find any prognostic role for either Goseki or other histological classification systems, patients' age, sex or Siewert topographical classification. According to Goseki classification, it should be noted that somewhat inconsistent results were shown by the two independent studies finding significant differences in survival of patients with gastric carcinoma (Martin *et al*, 1994; Songun *et al*, 1999). The 5-year survival rates or median survival times varied according to Goseki grades in different ways in the two studies: Martin *et al* (1994) observed longer survival in Goseki grade III when compared with grade IV patients, while the contrary was found by Songun *et al* (1999). It is also noteworthy that Songun *et al* (1999) found a weak association between Goseki classification and survival of patients with gastric carcinoma, with a hazard ratio of 1.5 for Goseki stage III *vs* I+IV, whereas the risk of dying according to TNM stage reached a peak of 8.2 for patients in the most unfavourable stage. Therefore, although our study cannot be considered conclusive because of its small size and further research is mandatory, when taken together the present findings do not support the hypothesis that relevant differences in survival exist in patients with cardia adenocarcinoma according to Goseki classification.

With respect to the other prognostic factors investigated, our study shows that, in the absence of distant metastases, only lymph node involvement (N) is a statistically significant prognostic factor for cardia carcinoma in patients undergoing curative surgery, when taking account of the other factors as well. Deep wall invasion (local infiltration depth, T) was no longer significant after considering lymph node involvement, contrary to evidence from another study showing local infiltration depth but not node involvement as predictive of survival (Jakl *et al*, 1995). However, it is well known that these two factors are strongly related and therefore it is difficult to disentangle the role of each of them. Accordingly, histological grading was associated with other prognostic indexes, particularly lymph node positivity, since none of the 9 patients with well-differentiated carcinoma had node positivity, in contrast with 74.4% of the 43 with poorly differentiated types. We also found that the Siewert topographical classification was of no prognostic value, although only 5 subjects were classified as types I, thus preventing proper comparisons between this group and the others.

In conclusion, we found no prognostic role for Goseki or other traditional histological classification systems or for Siewert topographical classification; however, we did confirm the relevance of the TNM staging system as the most important predictor of survival in patients with adenocarcinoma of the cardia.
